# Copy number variation of the *CCDC39* gene is associated with growth traits in Chinese cattle

**DOI:** 10.1002/vms3.712

**Published:** 2022-03-02

**Authors:** Linyong Hu, Junjian Yu, Rong Huang, Peng Yang, Zijing Zhang, Yanan Chai, Qiaoting Shi, Fuying Chen, Xian Liu, Zhiming Li, Baorui Ru, Eryao Wang, Chuzhao Lei, Wei Peng, Yongzhen Huang

**Affiliations:** ^1^ Key Laboratory of Adaptation and Evolution of Plateau Biota, Northwest Institute of Plateau Biology Chinese Academy of Sciences Xining Qinghai People's Republic of China; ^2^ College of Animal Science and Technology Northwest A&F University Yangling Qinghai People's Republic of China; ^3^ Qinghai Academy of Animal Science and Veterinary Medicine Qinghai University Xining Qinghai People's Republic of China; ^4^ Institute of Animal Husbandry and Veterinary Science Henan Academy of Agricultural Sciences Zhengzhou Henan People's Republic of China; ^5^ Henan Provincial Animal Husbandry General Station Zhengzhou Henan People's Republic of China

**Keywords:** association, *CCDC39* gene, Chinese beef cattle, copy number variations (CNV), growth trait, mRNA expression

## Abstract

**Background:**

Copy number variation (CNV) has become an essential part of genetic structural variation. Coiled‐coil domain containing 39 (*CCDC39*) is a gene that related to the growth and development of organs and tissues. It is identified that it has a CNV region by animal genome resequencing.

**Objective:**

In this study, we detected the phenotypic traits and different distributions of *CCDC39* gene copy numbers in five Chinese cattle breeds (Qinchuan (QC) cattle, Yunling (YL) cattle, Xianan (XN) cattle, Pinan (PN) cattle and Jiaxian (JX) cattle).

**Methods:**

Five hundred and six cattle were randomly selected for CNV distribution detection. Blood samples were taken and genomic DNA was extracted. Different tissues were obtained from adult (*n* = 3) XN cattle, including heart, liver, kidney, skeletal muscle and lung. The genome qPCR experiment was performed with SYBR Green in triplicate. CDNA qPCR was used to detect the expression level of *CCDC39* in different tissues and varieties. Using SPSS v20.0 software, the relationship between *CCDC39* CNV and the growth traits of PN, XN, QC, NY and YL cattle breeds was analyzed by one‐way analysis of variance (ANOVA).

**Results:**

The results showed that the expression of *CCDC39* in lung was higher than that in other tissues. The expression in liver and kidney was similar, but the expression in heart and muscle was less. It can be seen that the duplication type of QC cattle *CCDC39* CNV is higher than the deletion or normal in the height at hip cross. The normal type of PN cattle in body length and hip width was better than duplication and deletion (*p *< 0.05). In XN cattle, the deletion type of CNV had superior growth characteristics in heart girth and cannon bone circumference compared with the duplication type and the normal type (*p *< 0.05).

**Conclusion:**

The study revealed a significant association between CNV of *CCDC39* gene and growth traits in different Chinese cattle breeds.

## Graphical abstract

We detected the different distributions of *CCDC39* gene copy numbers in five Chinese cattle breeds. We also detected the expression situation of *CCDC39* gene in cattle. The study revealed the significant correlation between CNVs of *CCDC39* gene and growth traits in different Chinese cattle breeds.

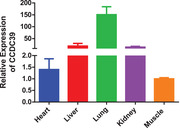

## 1 INTRODUCTION

Chinese yellow cattle is an important resource of beef cattle (Barbato et al., 2020). Nowadays, using the means of molecular breeding to breed cattle with great growth traits has become a significant method in animal breeding (Mohammadabadi, 2021; Norouzy et al., 2005; Shamsalddini et al., 2016). As the carrier of genetic information, genomic DNA plays an important part in the formation of phenotypic traits and the genetic differences between individuals mainly caused by genomic variation (Mohammadi et al., 2009; Mohammadabadi et al., 2007, 2011; Mohammadabadi, 2020). Copy number variations (CNVs) present in the form of deletion or insertions of large‐scale fragments in genomic DNA, and as a newly discovered genomic structural variation type, they are defined ranging from 50 bp to 1 Mb (Sohrabi et al., 2018). The type includes the duplication and deletion of copy number (Feuk et al., 2006) based on the log_2_2^–ΔΔCt^ values. Many studies have discussed the variants of gene in chicken and analyzed the association between them (W. Li et al., 2019). It was discovered that different types of gene's copy number in the cattle influenced the growth traits differently such as CNV of *LEPR* gene (Shi et al., 2016). The CNVs of *LEPR* gene were significantly associated with phenotypic traits of Nanyang cattle, and individuals with copy number duplication and/or normal had better phenotypic traits than the deletion groups (*p* < 0.05) (Shi et al., 2016).

By searching from NCBI, we know that coiled‐coil domain containing 39 (*CCDC39*) is located on cattle chromosome 1 and contains 20 exons. In human terms, the protein encoded by *CCDC39* gene is related to the motility of cilia and flagella. The encoded protein is essential for the assembly of dynein regulatory and inner dynein arm complexes, which regulate ciliary beat. The gene broadly express in human's testis, lymph node and 23 other tissues. Mutations in this gene are a cause of primary ciliary dyskinesia, and the gene is essential for assembly of inner dynein arms and the dynein regulatory complex (Merveille et al., 2011). Recently, a novel animal model of hydrocephalus due to a genetic mutation in the *CCDC39* gene has been developed in mice (Goulding et al., 2020). It might be an important candidate gene in animal breeding that can be used to improve the relevant economic traits of bovine growth. For humans, the defects in this gene are a cause of primary ciliary dyskinesia type 14 (CILD14) (RefSeq, July 2011). The pre‐sequencing results showed that there was a CNV region on the *CCDC39* gene (Huang et al., 2021). However, the gene's tissues expression of Chinese cattle has not been ascertained. In this study, we identified the expression of *CCDC39* gene in Chinese cattle by qPCR and ascertained the CNV region exists. Finally, the study aims to search the influence of CNVs of *CCDC39* gene to cattle growth traits.

## 2 MATERIALS AND METHODS

### 2.1 Animals and growth traits measurements

Five Chinese beef cattle (Table S1) breeds were involved in this study to detect the intergroup distribution of *CCDC39* gene CNV: Yunling cattle (YL, *n* = 118, Kunming city, Yunnan Province, China), Qinchuan cattle (QC, *n* = 93, Fufeng county, Shanxi Province, China), Jiaxian cattle (JX, *n* = 67, Jiaxian county, Henan Province, China), Pinan cattle (PN, *n* = 123, Xinye county, Henan Province, China) and Xianan cattle (XN, *n* = 105, Biyang county, Henan Province, China). Five hundred and six cattle were randomly selected for CNV distribution detection (Table S1). In addition, XN cattle breeds were enrolled in order to detect the mRNA expression level of *CCDC39* gene. All the individuals were randomly selected as test cases. In addition, individuals of each breed are selected from the same farm. Blood samples were collected from the ears of cattle, and genomic DNA was isolated from white blood cells by phenol‐chloroform extraction (Abadi et al., 2008). The phenotypic data of five breeds of the age 2–3 years old, including growth traits such as weight, height, body length, chest circumference, bone width and average daily duplication, were recorded for further association analysis. The phenotypic data of JX cattle were provided by Henan Pingdingshan Livestock and Poultry Improved Breeding Co., Ltd, and the other data were measured and recorded by our laboratory. All aforesaid cattle (without genetic relationships) were female and weaning‐to‐slaughtered, corn silage freely‐fed.

### 2.2 Sample collected, genomic DNA and total RNA extraction

Blood samples were taken and genomic DNA was extracted following the procedure described by Sonstegard et al. (2000). Adult XN cattle were selected for tissue sampling. As the first beef cattle breed in China, XN cattle is representative. In addition, our laboratory has previously compared several bovine expression profiles of other genes and found the same trend. Finally, we chose the XN cattle. None of the animals showed any signs of ill health. Different tissues were obtained from adult (*n* = 3) XN cattle, including heart, liver, kidney, skeletal muscle and lung. These tissues were rapidly frozen in liquid nitrogen and stored at −80°C for subsequent use. Total RNA was isolated using Trizol reagent (Takara, Dalian, China) according to manufacturer's instructions. The mass of ribosomal RNA bands was determined by agar‐gel electrophoresis and spectrophotometry. The first strand of cDNA was synthesized with 1 μg total RNA using a cDNA synthesis kit (Takara).

### 2.3 Copy number analysis of *CCDC39* gene

In this study, we studied the relative copy number of bovine *CCDC39* gene. Bovine basic transcription factor 3 (*BTF3*) was selected as the internal reference gene because there was neither CNV nor fragment replication in the *BTF3* genomic variation database (Bickhart et al., 2012). The *CCDC39* gene copy number was confirmed based on the hypothesis that there were two DNA fragments in the calibrated animal. The genome qPCR experiment was performed with SYBR Green in triplicate. A total of 10.0 μl reaction mixtures contained 1 μl of DNA, 5.00 μl SYBR Premix Ex Taq TM II (TaKaRa) and 1 μl of primers. Thermal cycling conditions consisted of one cycle of 10 min at 95°C, followed by 39 cycles of 15 s at 95°C, 60 s at 60°C and 5 s at 65°C.

#### 2.3.1 Expression profiling of *CCDC39* gene

cDNA qPCR was used to detect the expression level of *CCDC39* in different tissues and varieties in CFX96TM real‐time detection system (Bio‐Rad, Hercules, CA, USA) (Table S2). The relative expression of this gene was normalized to the expression of bovine β‐actin (Table S3). The qPCR experiment was conducted using *CCDC39* and β‐actin gene‐specific primer pairs. The qPCR system and procedures are the same as above. Using gene expression macro software (application of biological systems, Life Technology, Carlsbad, California, USA) and quantitatively *CCDC39* gene expression level by adopting optimized compare circulating threshold (Ct) (ΔΔCt) value method, usually designated as 2^–ΔΔCt^. All experiments were repeated three times and the mean of the intensity ratio ± SD was plotted.

### 2.4 Statistical analysis

In all the qPCR experiments, the analysis results were analyzed by Ct, which represented the mean ± SD of the three independent samples in the study (W. Li et al., 2020). Gene expression abundance performed by 2^–ΔΔCt^, ΔCt = Ct _target gene_ − Ct _reference genes_ (J. W. Xu et al., 2019). Copy number types are divided into three types, duplication (>0.5), deletion (← 0.5) and normal (≤| ± 0.5|) in our study. The calculation is based on log_2_2^–ΔΔCt^ (–ΔΔCt) relative to the reference sample. The function of *CCDC39* gene in mRNA expression was analyzed by using Excel 2010 and prism 6.0 software. Using SPSS v20.0 software (SPSS, Inc., Chicago, IL, USA), the relationship between *CCDC39* CNV and the growth traits of PN, XN, QC, NY and YL cattle breeds was analyzed by one‐way analysis of variance (ANOVA). In the data processing, according to the different factors affecting the body size traits, considering age and genetic effects, the fixed model was used for analysis, and simplified according to the actual situation. To analyze the effect on phenotypic traits, we used the following model: *Y_ijk_* = *μ* + *A_i_* + *G_j_* + *e_ijk_*, where *Y_ijk_* is observed for growth traits, *μ* is the population mean for growth traits, *A_i_* is the effect due to the age of *ith*, *G_j_* is the effect due to CNV type of each point and *e_ijk_* is the random residual (Z. Xu et al., 2020; Yang et al., 2020).

## 3 RESULTS

### 3.1 Expression profiling of *CCDC39* gene

**FIGURE 1:**
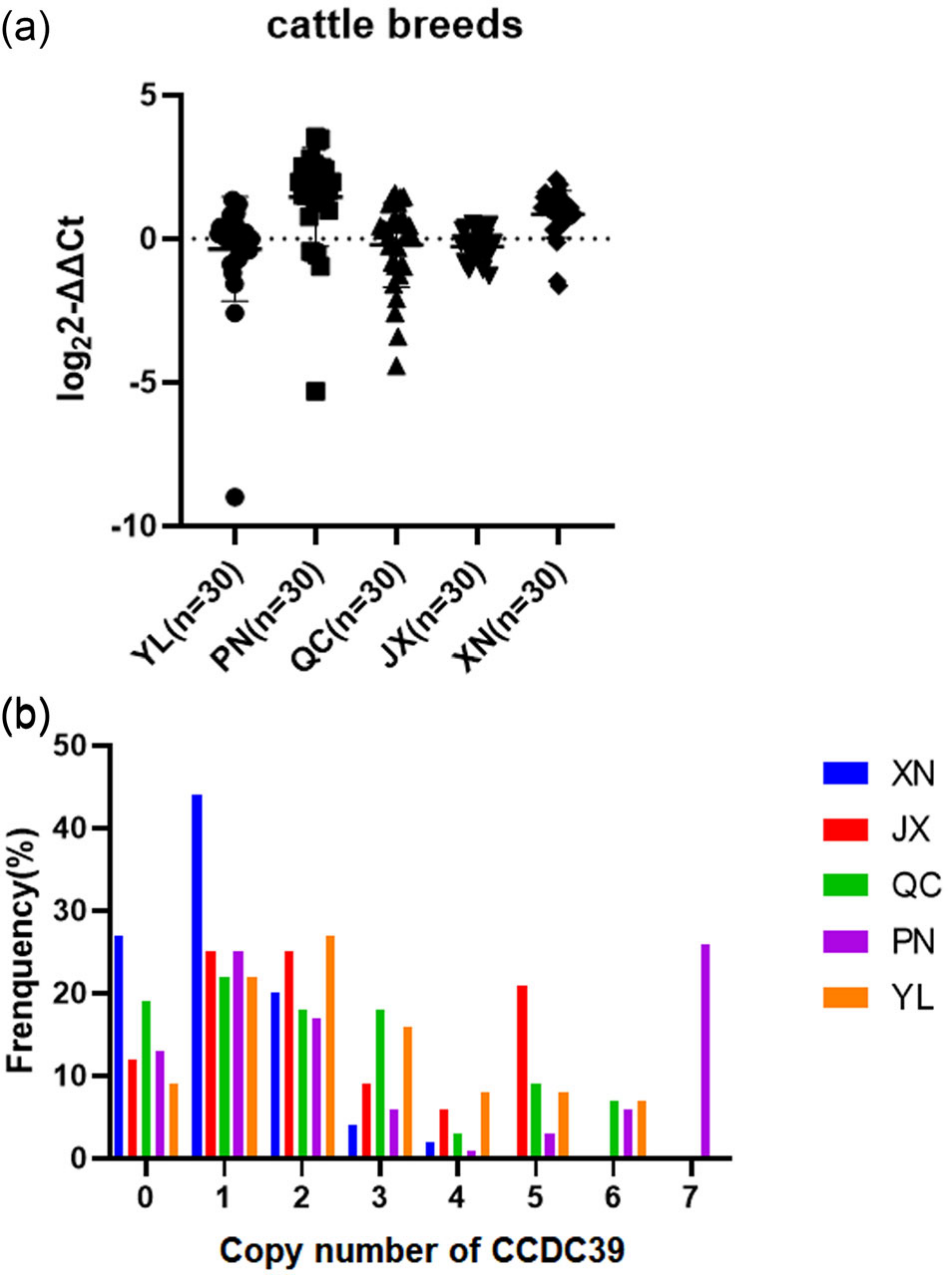
(a and b) The position and distribution of *CCDC39* gene copy number variation in cattle population in the invention. Abbreviations: JX, Jiaxian red cattle; PN, Pinan cattle; QC, Quinchuan cattle; XN, Xianan cattle; YL, Yunling cattle

As a protein‐coding gene, *CCDC39* affects the growth and differentiation of lung, heart, muscle and other tissues. Here, we present the expression profile of *CCDC39* gene in five different tissues of XN cattle (Figure 1). The results showed that the expression of *CCDC39* in lung was higher than that in other tissues. The expression in liver and kidney was similar, but the expression in heart and muscle was less (Figure 1).

### 3.2 The distribution of *CCDC39* gene copy number in five cattle breeds

**FIGURE 2:**
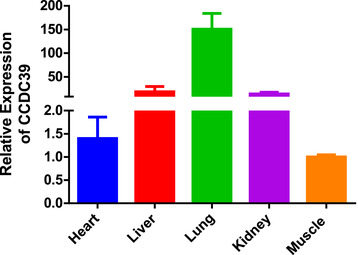
Expression profiling of *CCDC39* gene in cattle breed

In order to study CNV distribution of *CCDC39* in different cattle breeds, five cattle breeds including QC, PN, XN, YL and JX were selected. Based on the log_2_2^–ΔΔCt^ values, the CNV types were classified as duplication (>0.5), deletion (<0.5) and normal (≤|± 0.5|). As shown in Figure 2, the distribution of *CCDC39* CNV shows that copy number duplication is more dominant than deletion and normal in PN and XN cattle. It is worth noting that the relative copy number of *CCDC39* gene in QC group is highly variable compared with other test groups (Figure 2a).

In addition, Figure 2b shows the frequency of *CCDC39* relative copy number subjects in five cattle breeds. As can be seen from the figure, cattle with CN = 1 have a higher frequency in CNV than cattle with other copy numbers. The CN < 2 frequency of CNV in XN was higher than that of other breeds, indicating that copy number reduction was the main variation of XN cattle. All varieties showed variation types of duplication and deletion, indicating that variation existed in CNV region of *CCDC39* gene.

### 3.3 Association analysis between *CCDC39* CNV and growth traits in five cattle breeds

In recent years, many studies have reported that CNVs are associated with human height, livestock growth and carcass traits (X. Li et al., 2010). In this study, the association between *CCDC39* gene copy number type and growth traits of five cattle breeds (XN, PN, JX, QC and YL) was analyzed by one‐way ANOVA. In the statistical model, the *CCDC39* copy number types of the five cattle breeds were normalized to the above reference samples. Correspondingly, the three copy numbers (duplication, deletion and normal) are ≥3, <2 and 2 copies. It can be seen from Table 1 that the duplication type of QC cattle *CCDC39* CNV is better than the deletion/normal in the height at hip cross. The normal type of PN cattle in body length and hip width (Table 2) was better than duplication and deletion (*p *< 0.05). In XN cattle (Table 3), the deletion type of CNV had superior growth characteristics in heart girth and cannon bone circumference compared with the duplication type and the normal type (*p *< 0.05). Although the association is not significant, we can find that, in JX cattle (Table 4), the deletion type performed better than other types in some traits such as body length, and in YL cattle (Table 5), the normal type was better in traits such as body weight. The results showed that the CNV of *CCDC39* gene had a significant influence on the growth traits of cattle.

**TABLE 1:** Statistical association analysis of *CCDC39* gene copy number variation (CNV) with growth traits in Quinchuan cattle (QC) cattle

		CNV types (mean ± SE)			
Breed	Growth traits	Deletion (*n* = 39)	Normal (*n* = 15)	Duplication (*n* = 39)	*p‐*Value
Qinchuan	Body height (cm)	130.51 ± 0.96	126.27 ± 1.54	129.08 ± 0.96	0.068
	Height at hip cross (cm)	128.24 ± 0.93^a^	123.80 ± 1.49^b^	126.14 ± 0.93^a^ ^b^	0.036^*^
	Body length (cm)	137.97 ± 1.89	138.27 ± 3.05	138.80 ± 1.89	0.953
	Heart girth (cm)	175.64 ± 3.96	180.40 ± 6.38	179.41 ± 3.96	0.733
	Chest width (cm)	38.31 ± 0.81	41.40 ± 1.30	38.72 ± 0.81	0.124
	Chest depth (cm)	64.17 ± 0.91	64.23 ± 1.46	64.92 ± 0.91	0.825
	Rump length (cm)	43.62 ± 0.66	44.27 ± 1.06	44.05 ± 0.66	0.836
	Hucklebone width (cm)	22.37 ± 0.67	23.80 ± 1.08	24.12 ± 0.67	0.172
	Hip width (cm)	42.85 ± 0.78	43.20 ± 1.26	42.59 ± 0.78	0.915
	Body weight (kg)	367.00 ± 15.52	382.86 ± 25.02	382.14 ± 15.52	0.754

*Notes*: Values with different superscripts (a and b) within the same row differ significantly at *p *< 0.05.

**TABLE 2:** Statistical association analysis of *CCDC39* gene copy number variation (CNV) with growth traits in Jiaxian (JX) cattle

		CNV types (mean ± SE)			
Breed	Growth traits	Deletion (*n* = 30)	Normal (*n* = 18)	Duplication (*n* = 19)	*p‐*Value
Jiaxian	Withers height(cm)	124.08 ± 1.18	123.17 ± 1.53	124.82 ± 1.49	0.741
	Body length (cm)	145.83 ± 1.97	140.06 ± 2.54	143.11 ± 2.47	0.203
	Heart girth (cm)	177.00 ± 2.40	170.50 ± 3.10	171.63 ± 3.10	0.188
	Hip width (cm)	45.70 ± 0.77	44.58 ± 1.00	44.75 ± 1.00	0.611
	Hucklebone width (cm)	22.00 ± 1.12	23.19 ± 1.44	24.55 ± 1.40	0.366
	Rump length (cm)	45.68 ± 0.87	45.97 ± 1.12	44.87 ± 1.09	0.759
	Height at hip cross (cm)	124.32 ± 1.12	124.03 ± 1.44	124.16 ± 1.40	0.987
	Height at sacrum (cm)	116.37 ± 1.03	116.94 ± 1.33	117.47 ± 1.30	0.797
	Chest depth (cm)	64.30 ± 1.41	61.61 ± 1.82	63.16 ± 1.77	0.508
	Chest width (cm)	38.82 ± 1.01	37.42 ± 1.3	38.42 ± 1.27	0.696
	Body weight (kg)	427.18 ± 16.08	365.66 ± 21.17	390.53 ± 21.17	0.067

**TABLE 3:** Statistical association analysis of *CCDC39* gene copy number variation (CNV) with growth traits in Pinan (PN) cattle

		CNV types (mean ± SE)			
Breed	Growth traits	Deletion (*n* = 47)	Normal (*n* = 22)	Duplication (*n* = 54)	*p‐*Value
Pinan	Withers height (cm)	124.70 ± 0.87	125.68 ± 1.28	126.28 ± 0.81	0.419
	Body length (cm)	146.62 ± 1.43^b^	151.45 ± 2.09^ab^	151.24 ± 1.33^a^	0.039^*^
	Height at hip cross (cm)	131.77 ± 0.84	133.23 ± 1.23	133.09 ± 0.78	0.444
	Heart girth (cm)	173.19 ± 1.88	176.55 ± 2.75	176.31 ± 1.76	0.414
	Hip width (cm)	46.68 ± 0.56^ab^	48.41 ± 0.81^a^	45.47 ± 0.52^b^	0.010*
	Rump length (cm)	48.72 ± 0.55	49.32 ± 0.81	49.30 ± 0.51	0.711

*Notes*: Values with different superscripts (a and b) within the same row differ significantly at *p *< 0.05.

**TABLE 4:** Statistical association analysis of *CCDC39* gene copy number variation (CNV) with growth traits in Yunling (YL) cattle

		CNV types (mean ± SE)			
Breed	Growth traits	Deletion (*n* = 38)	Normal (*n* = 34)	Duplication (*n* = 46)	*p‐*Value
Yunling	Body height (cm)	127.16 ± 0.77	128.21 ± 0.82	127.13 ± 0.70	0.550
	Height at hip cross (cm)	131.21 ± 0.88	132.41 ± 0.93	131.39 ± 0.80	0.603
	Body length (cm)	154.35 ± 1.36	157.50 ± 1.41	157.15 ± 1.22	0.200
	Heart girth (cm)	193.31 ± 1.36	194.09 ± 1.40	194.33 ± 1.21	0.848
	cannon bone circumference (cm)	18.08 ± 0.22	18.09 ± 0.23	18.35 ± 0.20	0.576
	Chest width (cm)	48.16 ± 0.75	48.53 ± 0.78	48.28 ± 0.67	0.942
	Chest depth (cm)	68.08 ± 0.98	69.91 ± 1.02	69.98 ± 0.88	0.293
	Hip width (cm)	56.05 ± 0.80	56.18 ± 0.83	57.15 ± 0.71	0.525
	Hucklebone width (cm)	22.03 ± 0.30	21.82 ± 0.32	21.80 ± 0.27	0.840
	Rump length (cm)	48.97 ± 0.55	50.56 ± 0.58	50.48 ± 0.50	0.076
	Body weight (kg)	540.11 ± 10.03	542.18 ± 10.83	555.85 ± 9.26	0.456

**TABLE 5:** Statistical association analysis of *CCDC39* gene copy number variation (CNV) with growth traits in Xianan (XN) cattle

		CNV types (mean ± SE)			
Breed	Growth traits	Deletion (*n* = 31)	Normal (*n* = 27)	Duplication (*n* = 47)	*p‐*Value
Xianan	Withers height (cm)	135.26 ± 0.99	135.67 ± 1.06	134.32 ± 0.80	0.558
	Height at hip cross (cm)	138.81 ± 0.73	138.11 ± 0.78	138.00 ± 0.59	0.672
	Body length (cm)	159.10 ± 1.29	159.33 ± 1.38	157.57 ± 1.05	0.509
	Heart girth (cm)	198.35 ± 1.66^a^	194.48 ± 1.73^ab^	194.26 ± 1.31^b^	0.004*
	cannon bone circumference (cm)	20.66 ± 0.30^a^	19.41 ± 0.32^b^	19.07 ± 0.24^b^	0.000*
	Body weight (kg)	559.00 ± 10.67	549.52 ± 11.43	535.81 ± 8.66	0.232

*Notes*: Values with different superscripts (a and b) within the same row differ significantly at *p *< 0.05.

## 4 DISCUSSION

CNV detection is an important part of genomic mutation detection, which affects the phenotypic changes of individual organisms through dose effect (Jeon et al., 2010; Wright et al., 2009). So far, many studies have shown that CNV can affect economic traits of animals and lay the foundation for molecular breeding, such as pigs (Stachowiak et al., 2017), cattle (Y. Xu et al., 2017), chickens (Gorla et al., 2017) and sheep (Ma et al., 2017). There are many methods to detect CNV, but qPCR method is simple and practical. In the past, it was usually detected by array‐based comparative genomic hybridization or sequencing method (Pinto et al., 2011). We used *CCDC39* candidate gene qPCR technology to detect the mRNA expression and CNV types of Chinese cattle population, and analyzed the morphology related to growth and development.

Based on the type detection of *CCDC39* gene copy number in five Chinese cattle breeds and the association analysis with growth traits, we found that there was variation in the relative copy number of *CCDC39* gene in different Chinese cattle breeds. From the difference of CNV frequency of five breeds, the increase of copy number of XN cattle was higher than that of other breeds. This may be due to differences in breeding conditions between different breeds of cattle (Lehnert et al., 2007). XN cattle is a breed of French Charolais cattle (male) and Nanyang cattle (female), while other cattle are mostly Chinese native breeds and belong to service cattle and beef cattle. In particular, QC cattle *CCDC39* had a significant difference in relative copy number, indicating that *CCDC39* CNV gene locus has a rich genetic copy number diversity, which may have a potential impact on the phenotype of different cultivar. We hypothesized that CNV in the bovine genome may occur in breeds with different patterns and may lead to breed differences, thus related to breed formation and adaptation.

The experimental results show that *CCDC39* CNV variation can indeed affect the growth characteristics of Chinese cattle significantly, such as heart girth, cannon bone circumference, body length, hip width and height at hip cross. We also detected the expression of *CCDC39* gene in different organs to assist us in analyzing the relationship between CNV of *CCDC39* gene and growth traits. The results showed that the expression of *CCDC39* in lung was higher than that in other tissues. Bovine *CCDC39* gene has high homology with human and has many biological functions. The mutations in *CCDC39* and CCDC40 are the major cause of primary ciliary dyskinesia with axonemal disorganization and absent inner dynein arms (Antony et al., 2013). These changes in biological functions of *CCDC39* will affect the growth and development of the organism, and may produce significant phenotypic differences through potential mechanisms. This method can be used in the future molecular marker breeding of cattle.

## 5 CONCLUSION

To sum up, we found and detected CNV of *CCDC39* gene in different cattle breeds in China. This variation has a significant effect on the growth character of cattle to a certain extent, which is confirmed by association analysis. The expression of *CCDC39* gene in Chinese bovine tissues was detected by mRNA level, revealing the important influence of *CCDC39* gene on bovine growth and development. Our study demonstrated for the first time the functional effects of *CCDC39* CNV in large populations and different breeds, in order to provide evidence for the great potential of CNV as a new molecular marker in cattle breeding.

## ANIMAL CARE

The protocols used in this study for the animals were recognized by the Faculty Animal Policy and Welfare Committee of Northwest A&F University (FAPWC‐NWAFU, protocol number, NWAFAC1008).

## CONFLICT OF INTEREST

The authors declare no conflict of interest.

## AUTHOR CONTRIBUTIONS

Conceptualization: Hu L, Yu J, Peng W, Huang Y; Datacuration: Hu L, Lei C, Huang Y; Formalanalysis: Yu J, Huang R; Fundingacquisition: Hu L, Peng W, Huang Y; Investigation: Lei C, Huang Y; Methodology: Yu J, Huang R, Yang P, Zhang Z; Projectadministration: Hu L, Peng W, Huang Y; Resources: Chai Y, Shi Q, Chen F, Liu Xian, Li Z, Ru B, Wang E, Lei C, Huang Y; Software: Yu J, Huang R, Yang P; Validation: Hu L, Yu J, Peng W, Huang Y; Writing‐original draft: Hu L, Yu J, Huang R; Writing – review & editing: Peng W, Huang Y.

### PEER REVIEW

The peer review history for this article is available at https://publons.com/publon/10.1002/vms3.712

## Supporting information

Table S1 The information of Chinese Yellow Cattle sample
Table S2 Primer information for qPCR
Table S3 Comparison of CNV overlapping *CCDC39* in this study with other cattle CNV‐related studies.
