# Supplementary material for: Copy number variation of the CCDC39 gene is associated with growth traits in Chinese cattle
Source: Vet Med Sci. 2022 Mar 2;8(2):917–24. doi: 10.1002/vms3.712 (PMC8959325; doi:10.1002/vms3.712)
Supplement: Supplementary file 1 — Table S1 The information of Chinese Yellow Cattle sample Table S2 Primer information for qPCR Table S3 Comparison of CNV overlapping CCDC39 in this study with other cattle CNV‐related studies. [file VMS3-8-917-s001.docx]

**Table S1 The information of Chinese Yellow Cattle sample**

| Breeds | Number | Origin | Growth traits |
| --- | --- | --- | --- |
| Qinchuan cattle，QC | 93 | Qinchuan cattle breeding center of Fufeng County, Baoji City, Shaanxi Province | 24-36 months old |
| Jiaxian Red cattle，JX | 67 | Jiaxian Red Cattle Breeding Center, Pingdingshan City, Henan Province | 24-36 months old |
| Pinan cattle， PN | 123 | Core breeding area of Pinan cattle in Xinye County, Nanyang City, Henan Province | 24-36 months old |
| Yunlin cattle，YL | 118 | Academy of grassland zoology, Xiaoshao Township, Kunming City, Yunnan Province | 24-36 months old |
| Xianan cattle，XN | 105 | Xianan cattle Technology Limited Company in Miyang County, Zhumadian City, Henan Province | 24-36 months old |
| Total | 506 |  |  |

**Table S2 Primer information for QPCR**

| Gene | Primer pairs sequences(5’-3’) | Amplification length(bp) |
| --- | --- | --- |
| *CCDC39*  *BTF3* | F：5’- AGCTCAGGTGACTAGGGACA -3’  R：5’- TGGGCATCATCAGGGAAAGAG -3’  F：5’-AACCAGGAGAAACTCGCCAA-3’  R：5’-TTCGGTGAAATGCCCTCTCG-3’ | 146 bp    166 bp |

**Table S3** Comparison of CNV overlapping *CCDC39* in this study with other cattle CNV-related studies.

| Gene | chromosome | CNV position | size |
| --- | --- | --- | --- |
| *EIF4A2* | 1 | 81,347,201-81,351,200 | 4000 |
| *USP16* | 1 | 6,544,001-6,547,600 | 3,600 |
| *ZNF639* | 1 | 88,724,801-88,728,400 | 3600 |
| *ACTRT3* | 1 | 98,763,601-98,771,200 | 7600 |
| *CCDC39* | 1 | 87,264,801-87,269,200 | 4400 |
